# Braille in the Sighted: Teaching Tactile Reading to Sighted Adults

**DOI:** 10.1371/journal.pone.0155394

**Published:** 2016-05-17

**Authors:** Łukasz Bola, Katarzyna Siuda-Krzywicka, Małgorzata Paplińska, Ewa Sumera, Paweł Hańczur, Marcin Szwed

**Affiliations:** 1 Department of Psychology, Jagiellonian University, Krakow, Poland; 2 Laboratory of Brain Imaging, Neurobiology Center, Nencki Institute of Experimental Biology, Warsaw, Poland; 3 INSERM U 1127, CNRS UMR 7225, Sorbonne Universités, and Université Pierre et Marie Curie-Paris 6, UMR S 1127, Institut du Cerveau et de la Moelle épinière (ICM), Paris, France; 4 Academy of Special Education in Warsaw, Warsaw, Poland; 5 Institute for the Blind and Partially Sighted Children in Krakow, Krakow, Poland; 6 Electrical Drive Division, Institute of Control and Industrial Electronics, Warsaw University of Technology, Warsaw, Poland; University of Chicago, UNITED STATES

## Abstract

Blind people are known to have superior perceptual abilities in their remaining senses. Several studies suggest that these enhancements are dependent on the specific experience of blind individuals, who use those remaining senses more than sighted subjects. In line with this view, sighted subjects, when trained, are able to significantly progress in relatively simple tactile tasks. However, the case of complex tactile tasks is less obvious, as some studies suggest that visual deprivation itself could confer large advantages in learning them. It remains unclear to what extent those complex skills, such as braille reading, can be learnt by sighted subjects. Here we enrolled twenty-nine sighted adults, mostly braille teachers and educators, in a 9-month braille reading course. At the beginning of the course, all subjects were naive in tactile braille reading. After the course, almost all were able to read whole braille words at a mean speed of 6 words-per-minute. Subjects with low tactile acuity did not differ significantly in braille reading speed from the rest of the group, indicating that low tactile acuity is not a limiting factor for learning braille, at least at this early stage of learning. Our study shows that most sighted adults can learn whole-word braille reading, given the right method and a considerable amount of motivation. The adult sensorimotor system can thus adapt, to some level, to very complex tactile tasks without visual deprivation. The pace of learning in our group was comparable to congenitally and early blind children learning braille in primary school, which suggests that the blind’s mastery of complex tactile tasks can, to a large extent, be explained by experience-dependent mechanisms.

## Introduction

A growing number of studies show that in sensory deprivation, perceptual abilities in the remaining senses become enhanced. When compared with sighted subjects, the blind show superior performance in specific tactile tasks [1–7] and auditory tasks [8,9]. Deaf people, in turn, show enhanced perception of peripheral visual stimuli [10]. These behavioral effects are associated with a potentially adaptive neural reorganization. For example, it was shown that large parts of the deprived sensory cortex are taken over by other senses, such as touch or hearing in the blind and vision in the deaf [11,12].

Blind people are particularly able to excel in remarkably complex tactile tasks, for example braille reading [13]. It remains unclear to what extent braille reading can be learnt without visual deprivation. In the case of relatively simple tactile tasks, such as discriminating grating orientation, several studies show that the blind’s superior performance can be explained by experience-dependent mechanisms, as blind subjects have more experience in using touch in their everyday lives [3,6]. In line with these results, sighted subjects are able to significantly progress in simple tactile tasks through training [14–18]. Braille reading, however, is a complex skill and prolonged visual deprivation is thought to greatly enhance its acquisition. In blind people, the onset of blindness largely determines proficiency in braille reading in adulthood. Congenitally and early blind individuals are able to achieve braille reading speed of 80–120 words-per-minute and more [13,19–21], whereas late-blind people usually read two to three times slower and many of them struggle to learn braille at all [4,13,20,22]. In sighted subjects, several days of blindfolding was shown to have beneficial effect on learning to recognize single braille characters [23,24]. In fact, children with residual sight are sometimes blindfolded to speed up the braille learning process (see braille teachers reports cited in: [25]). Based on all the above evidence, some researchers claim that “assessing the effects of tactile reading by training sighted people to read Braille is prohibitive” [26]. However, to our knowledge no study has systematically tested to what extent whole-word braille reading can be taught to sighted subjects with normal visual input. This may potentially revise the current view of perceptual capabilities of the human tactile system and inform in what sensory modality braille should be taught to teachers of visually impaired students.

Here we enrolled sighted adults in a 9-month braille reading course. We show that most sighted adults can learn whole-word braille reading, given the right method and motivation. The pace of braille acquisition in our sighted subjects was comparable to that of blind children learning braille reading in school [27–29]. Thus, our results might suggest that experience-dependent mechanisms largely account for the blind’s mastery in complex tactile tasks.

## Methods

### Subjects

Thirty-six individuals initially took part in the study (32 females, 4 males; median age = 27, range = 22–49). All were right-handed, fluent in Polish and had normal or corrected to normal vision. They were either braille teachers/educators (16 subjects), special education studies students specializing in blindness and related disabilities (15 subjects), or close relatives of blind people (5 subjects). Thirty-three participants reported that they know how to read visually presented braille but no one read it tactually (see Results).

For a variety of reasons (lack of time, personal reasons, loss of interest) 7 subjects resigned from the study. Twenty-nine subjects completed the course (26 females, 3 males; median age = 27, range = 22–49; 14 braille teachers/educators, 11 special education studies’ students, 4 relatives of blind people). Among the latter, 4 subjects did not attend some testing sessions, which resulted in 5 missing data points out of 232 (see Data Analysis section for details).

The study was approved by the local ethical committee (*Komisja ds*. *Etyki Badań Naukowych*) at the Jagiellonian University, Kraków. A written consent was obtained from each participant.

### Outline of the study and the braille course

**Fig 1:**
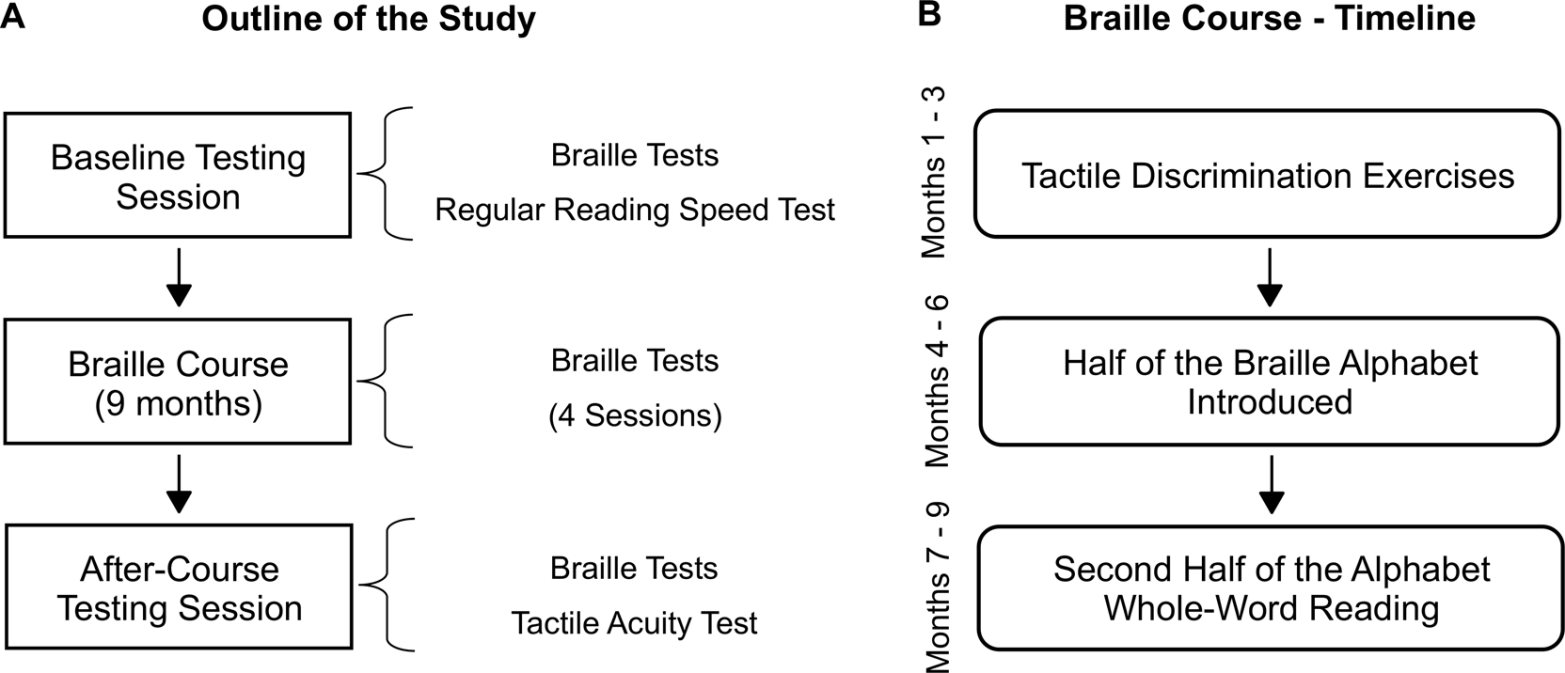
Outline of the study and timeline of the braille course. (A) Sighted adults underwent a 9-month braille course. They were tested behaviorally in the baseline testing session, four times during the course (i.e., in the 5^th^, 6^th^, 7^th^ and 8^th^ month of the course) and after the course. (B) The braille course consisted of three stages: tactile discrimination exercises, introducing half of the braille alphabet and learning the remaining half/whole-word reading.

An outline of the study is shown in Fig 1A and 1B. The course lasted 9 months. It included some pre-existing material from Polish [30] and English [31] textbooks, yet it created a new, original curriculum, tailored to sighted subjects’ needs. The course was designed by two experienced braille teachers (MP and ES, co-authors of this article). We took particular care not to overwhelm our subjects and introduced new material/skills gradually. Thus, during the first stage (3 months), subjects practiced only tactile discrimination. During the second stage (3 months) we gradually introduced half of the Polish braille alphabet (i.e., a, o, y, e, i, t, s, k, d, p, c, ł, m, u, l, b). Those letters were high-frequency, relatively easy to read in braille and sufficient to form simple sentences. The rest of the Polish braille alphabet (i.e., z, r, w, n, ć, ó, g, j, ś, ż, h, ą, ę, f, ź) was introduced and gradually learned only in the third stage (Fig 1B). All subjects were instructed to read with their index fingers. The right index finger (reading finger) was predominantly used for parsing braille words and recognizing braille characters, whereas the left index finger was used for navigation on the braille sheet.

The course relied primarily on participants’ individual work. Each month, they were given 30 exercises, each on a single sheet. They were asked to complete one exercise per day. Each exercise was performed blindfolded and then checked visually. The daily workload, as reported by the subjects, varied from 10 to 30 minutes. Once a month a group meeting was organized, during which participants were given a subsequent set of braille exercises and could resolve potential concerns with an expert braille teacher.

### Braille reading tests

The subjects’ braille reading speed was tested 6 times—at the beginning of the course (baseline), and once per month, starting in the fifth month (Fig 1A). Blindfolded participants were asked to read as many letters as possible aloud in 60 seconds time. Next, the procedure was repeated for words. Words were 3–6 letters long, and their frequency was higher than 1 per million, according to the SUBTLEX-PL database [32]. Stimuli were printed on a single braille sheet. In each testing session, a new set of words was used, and the order of letters was shuffled. The distribution of words' frequencies was similar across all versions of the test. Because we expected word length to have a significant impact on reading speed [33], the word lists were ordered such that on each version of the test, words of specific length appeared in exactly the same order (e.g., a 3-letter word was followed by a 5-letter word, and so on). Test words were composed only of the 16 letters introduced during the 2^nd^ stage of the course (see above).

### Tactile acuity test

At the end of the course (i.e., in the ninth month), we measured subjects’ final tactile acuity, as we expected it to be associated with their braille reading fluency (Fig 1A; [7,33]). The Grating Orientation Test [34] was used according to the standard procedure described by Van Boven et al. [3]. The test uses 8 plastic gratings with groove widths measuring 3.00, 2.00, 1.50, 1.25, 1.00, 0.75, 0.50 or 0.35 mm (JVP Domes, Stoelting Co., Wood Dale, IL). The test sites were the distal pads of the index finger (i.e., reading finger–see “outline of the study and the braille course”) and the middle finger of the subjects’ right hand. Subjects were seated in a quiet room and blindfolded. The hand was placed in a supinated position and tested fingers were immobilized using adhesive tape applied to nails. For each trial, the grating was applied along or perpendicular to the long axis of the finger and participants were asked to identify grating orientation (two-alternative forced choice paradigm). Gratings were applied manually by an experimenter, with moderate force judged to be comfortable by the subjects, and held for approximately 1.5 seconds. Subjects were required to keep the tested fingers stationary, which was visually inspected by the experimenter, and all trials in which movement between the skin and the grating occurred were rejected. 20 trials were administered for each grating, starting from the easiest one (i.e., the grating with grove width measuring 3.00 mm), and the test was terminated when the subject reached a chance level (50% correct responses). For each subject and tested finger, we set an individual grating orientation threshold by interpolating (using linear interpolation) the grating resolution at which 75% of responses would be correct.

### Silent reading speed in regular alphabet (Latin alphabet)

In addition to braille reading tests, subjects performed a test of visual reading speed in the regular alphabet, to relate their typical reading speed to their braille performance. The passage from a Polish book “Farsa Panny Heni” by Maria Rodziewiczówna was used. The passage consisted of 400 words. Subjects were asked to read the text silently, as fast and carefully as possible, and press a button right after they finished. Next, a paper-pencil test was administered to verify the passage comprehension. The test included 10 multiple-choice questions with three answer options. The total reading time in the reading speed test and accuracy in the comprehension test were collected as dependent measures. All subjects reported that, before the testing session, they had not known the book and the passage used for the test.

### Data analysis

All analyses were performed using SPSS 22 (SPSS Inc., Chicago, USA). To test for a general increase in braille reading speed, a repeated-measures ANOVA was performed on the number of words read in each testing session. Two subjects were excluded from the analysis, due to missing data from either the third or the fifth testing session. Twenty-seven subjects attended all six testing sessions and were included in the ANOVA. Subsequently, paired t-tests were used to compare word and letter reading speeds in the baseline session with the word and letter reading speeds in each of the following testing sessions. Comparisons including the third session and the fifth session were performed on 28 and 27 subjects, respectively, due to missing data. In the case of other testing sessions, all subjects were included.

In the case of the Grating Orientation Test, an individual grating orientation threshold was estimated for each subject and tested finger by interpolating (using linear interpolation) the grating resolution at which 75% of responses would be correct. Then, a paired t-test was used to compare grating orientation thresholds between the tested fingers (i.e., the index finger and the middle finger of the reading hand). Pearson correlation coefficients were calculated for grating orientation thresholds on tested fingers and final braille word reading speed. Subsequently, final braille word reading speeds in 5 subjects with the highest grating orientation threshold (i.e., the lowest tactile acuity) were compared with final braille word reading speeds in the rest of the group. Given the large disproportion in size of these two subgroups, a Mann-Whitney nonparametric test was applied for this comparison. All analyses including grating orientation threshold were performed on 28 subjects, as one subject did not attend the Grating Orientation Test.

We then tested whether the final braille reading speed was related to the subjects’ occupation or age. First, subjects were divided into three subgroups according to their demographic background (14 braille teachers/educators, 11 special education studies’ students, 4 relatives of blind people–see Subjects section) and final braille word reading speeds were compared between these subgroups. Given the large disproportion in size of the subgroups, a Kruskal-Wallis nonparametric test was used for this comparison. In the second analysis, final braille word reading speed was correlated with subjects’ age, using the Pearson correlation coefficient. Similarly, Pearson correlation was calculated for final braille word reading speed and the visual reading speed in regular (Latin) alphabet. The latter correlation was performed on 28 subjects, as one subject did not attend visual reading speed in regular alphabet test.

## Results

### Progress in braille reading

**Fig 2:**
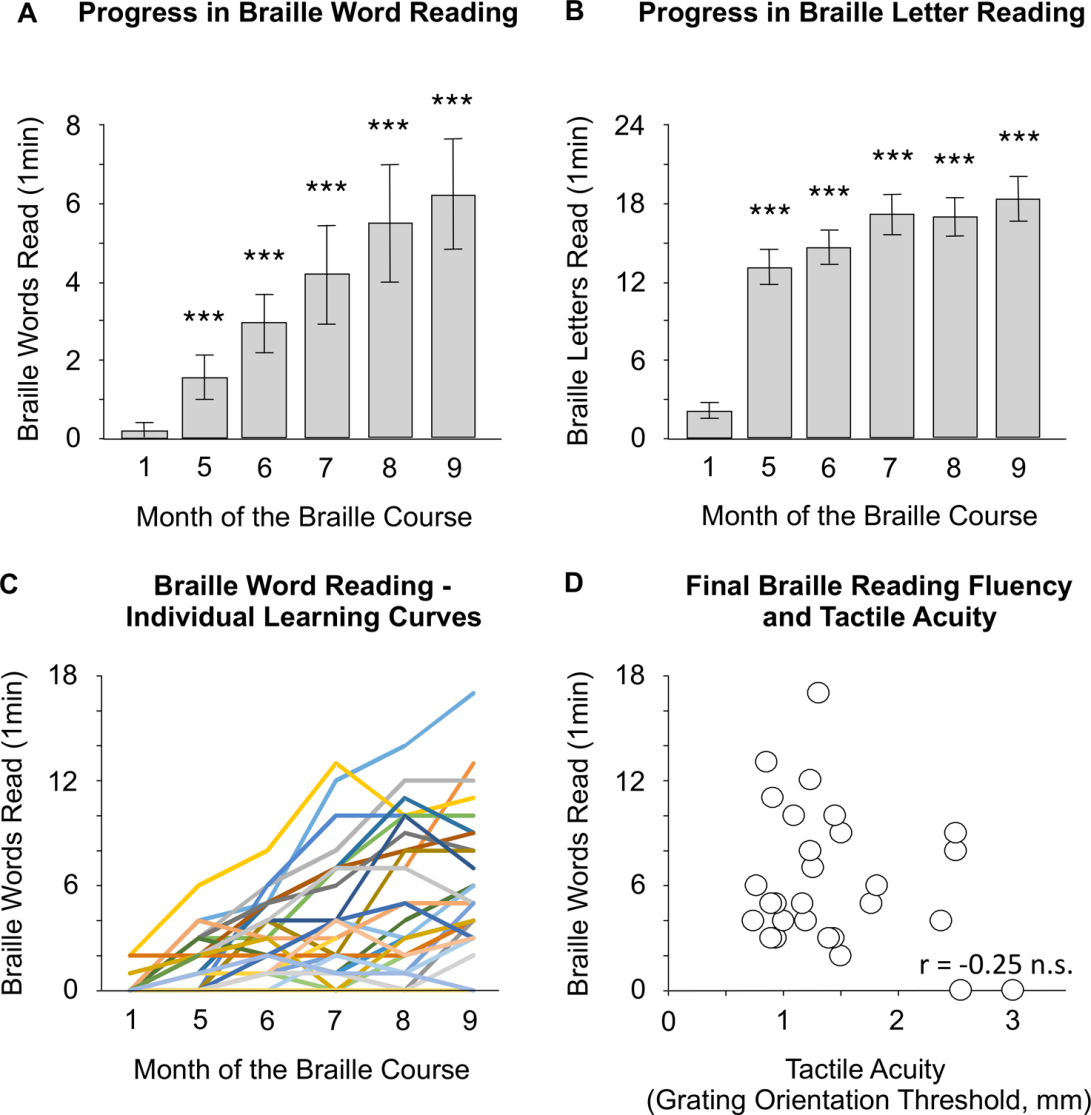
Sighted adults can learn whole-word braille reading. (A-B) Results of braille word reading test and braille letter reading test. Average number of braille words/letters read in one minute was plotted for each testing session. (C) Individual learning curves for braille word reading, plotted based on results of braille word reading test in each testing session. (D) Correlation between the tactile acuity (grating orientation threshold) at the end of the course and final braille word reading speed. Error bars represent 95% confidence intervals. Asterisks indicate a significant difference between the results of a specific testing session and a baseline testing session (*** p < 0.001).

At the onset of the study, 26 subjects (out of 29 included in the data analysis) were unable to read a single braille word in the 60 seconds allowed (Fig 2A). One subject managed to read one word and two subjects read two words. Moreover, most subjects could not recognize tactually even a single braille letter, (Fig 2B, median = 0, mean = 2.28, range = 0–7). Thus, at the beginning of the course, our subjects were utterly unable to read braille by touch.

The subjects quickly mastered individual braille letter recognition, and knew most letters by the second month of letter training/fifth month of the course (Fig 2B). They also gradually progressed in whole-word braille reading (Fig 2A). At the end of the course, subjects reached an average performance of 6.20 words-per-minute (WPM) (CI 95% = 4.79–7.62, range = 0–17) read aloud. To statistically test for an increase in the braille word reading speed, data from all six testing sessions were entered into a repeated-measures ANOVA (see Methods). This analysis confirmed that the increase in braille reading speed was highly significant (effect of testing session: F(5, 130) = 35.85, p < 0.001, η2 = 0.57). There were large inter-individual differences in tactile reading fluency (Fig 2C). The best reader in our group reached 17 WPM in the last testing session, while two subjects were unable to read even a single word.

### Relation to tactile acuity and demographic measures

Given that several studies show that tactile acuity level is correlated with braille reading speed in the blind [7,33], we expected to find a similar relationship in sighted subjects. To this aim, we measured subjects’ final tactile acuity with the Grating Orientation Test (see Methods). The mean grating orientation threshold for the reading finger was 1.43 mm (CI 95% = 1.19–1.67) and for the middle finger was 1.47 mm (CI 95% = 1.22–1.73). This difference was not significant (paired t-test, t(27) = 0.45, p > 0.250). We found no correlation between subjects’ final tactile acuity and final braille word reading fluency (Fig 2D; reading finger: r(26) = -0.25, p = 0.206; middle finger: r(26) = -0.26, p = 0.188). The final braille reading speed for 5 subjects with the lowest tactile acuity on the reading finger (mean grating orientation threshold = 2.59 mm, range = 2.40–3.00; mean final reading speed = 4.2 WPM, range = 0–9) was not significantly different from the rest of the group (mean grating orientation threshold = 1.18 mm, range = 0.75–1.78; mean final reading speed = 6.63 WPM, range = 2–17; Mann-Whitney U(26) = 38.5, p > 0.250).

We also tested whether progress in braille word reading was related to demographic variables (see Methods). First, subjects were divided into three subgroups according to their demographic background (14 braille teachers/educators, 11 special education studies’ students, 4 relatives of blind people–see Methods) and the final braille word reading speed was compared between these subgroups. However, no significant difference was found in this comparison (Kruskal-Wallis test, χ^2^(2, *N* = 29) = 1.50, p > 0.250). Thus, the subjects’ occupation was not related to their final braille word reading fluency. The subjects’ age was not correlated with their final braille word reading speed either (r(27) = 0.11, p > 0.250).

### Relation to regular reading

Subjects showed good comprehension of the text used for testing the visual reading speed in regular alphabet (mean accuracy in the comprehension test = 76%, CI 95% = 70–81). Their mean reading speed was 200 WPM (CI 95% = 182–218), which can be qualified as a standard adult visual reading speed [35]. Subjects’ visual reading speed was not correlated with final braille reading speed (r(26) = 0.03, p > 0.250).

## Discussion

Our study shows that most sighted adults can learn whole-word braille reading. To our knowledge, this is the first demonstration that adults with an intact visual system can learn such a complex tactile task, given the right motivation and method.

### Superior perception in the blind and its origin–visual deprivation hypothesis versus tactile experience hypothesis

Blind people outperform sighted individuals in specific auditory [8,9], olfactory [36,37] and tactile tasks [1–7]. Previous reports show that blind individuals have better tactile acuity [3–7], tactile 3-D shape discrimination [5], sense of tactile symmetry [1] and accelerated processing of tactile stimuli [2]. Moreover, blind people are able to master very complex tactile tasks, for example braille reading [13]. These enhancements in tactile perception are thought to be related to neural reorganization of the visual cortex, which, in blind people, is activated during tactile perception [1,11,12,38–41]. This activity has been shown to be essential for braille reading [42,43]. The somatosensory representation of the reading finger was shown to be enlarged in blind braille readers as well [44–46].

While the notion of superior tactile perception in blind people is well established, it is still controversial what is the cause of these enhancements. They might be caused by visual deprivation itself (visual deprivation hypothesis), or alternatively, by the extraordinarily rich tactile experience of blind people (tactile experience hypothesis). Several studies on tactile acuity, an important factor for various tactile tasks, support the visual deprivation hypothesis. Goldreich and Kanics [47], in particular, found that tactile acuity in all blind subjects was significantly superior to that of sighted subjects, but no difference in tactile acuity on the index finger was found between blind braille readers and non-readers. Moreover, tactile acuity in blind braille readers was not correlated with years of braille reading or daily amount of braille reading. This suggests that it is visual deprivation itself that increases tactile acuity, irrespectively of different levels of tactile experience in blind individuals. In line with this view, other experiments suggest that tactile acuity in sighted subjects can be improved through short-term (i.e., 45–90 minutes) blindfolding, even when no tactile training is applied [48,49]. However, two recent, extensive studies failed to confirm these observations [50,51]. Furthermore, several days of blindfolding was shown to be beneficial for learning to recognize single braille characters [23,24].

While the above-mentioned results might support the visual deprivation hypothesis, other experiments suggest that the blind’s superior tactile perception might be largely explained by experience-dependent mechanisms, namely their rich tactile experience. Under this view, the blind’s superior tactile perception is due to the fact that the blind have far more experience in using touch in their everyday lives than sighted individuals. In line with this notion, some studies suggest that tactile acuity is in fact modulated by the amount of tactile training in blind subjects. For example, Van Boven et al. [3] showed that blind braille readers have better tactile acuity on the preferred reading finger, which is extensively trained during braille reading, than on non-reading fingers. In a particularly informative study, Wong et al. [6] demonstrated that blind people outperform sighted individuals when tactile acuity is measured on fingers, but no difference between the blind and the sighted is observed when tactile acuity is measured on the lips, where tactile experience is supposed to be similar among these two populations. The authors also showed that proficient blind braille readers have better tactile acuity on the index finger preferably used for reading when compared to their opposite index finger or to tactile acuity in blind non-readers. Tactile acuity on the reading finger in blind braille readers was also correlated with their weekly reading time.

Further support for the tactile experience hypothesis comes from studies on sighted subjects. Several experiments show that sighted adults with normal visual input can significantly improve in relatively simple tactile tasks, such as discriminating orientation of gratings, frequency of tactile stimulation or recognizing single braille characters [14,15,17,18]. Moreover, neural reorganization observed in blind people was shown also in sighted subjects, following intensive training. Pleger et al. [52] and Hodzic et al. [53] show that several hours of tactile stimulation induce reorganization in the primary and the secondary somatosensory cortex in sighted adults. Several studies also demonstrate that the visual cortex in sighted subjects can be recruited for tactile tasks. Particularly, the lateral occipital complex, a high-level visual region in the ventral visual stream, was shown to be engaged in tactile recognition in sighted subjects [54–55]. In our own study, using the same subject group as described here, we demonstrated that another ventral visual region, the Visual Word Form Area, is critical for tactile braille learning in sighted adults [56]. Several studies show that also early visual areas of sighted subjects can be recruited for tactile discrimination tasks [57–59]. In summary, several results suggest that experience-dependent mechanisms might largely account for behavioral and neural differences among the blind and the sighted.

### Sighted adults can learn complex tactile tasks

Our study informs the debate about deprivation-based versus experience-dependent origin of the blind’s superior perceptual abilities. Previous experiments have shown that sighted subjects can significantly progress in relatively simple tactile tasks, for example discriminating the frequency of tactile stimulation [17], grating orientation [14,15,18] or recognizing single braille characters [16]. Here we show that most sighted adults are able to progress in a complex tactile task. Successful parsing of braille words requires remarkable perceptual and motor precision, and therefore poses a great demand on the human sensorimotor system, especially on the representation of the reading finger [4,13,25]. Our study thus demonstrates that the adult sensorimotor system can, to some level, adapt to very complex tactile tasks without visual deprivation.

While almost all subjects in our study were able to read whole braille words, we observed large inter-individual variance in braille learning outcomes. In line with this result, blind subjects also substantially differ in their braille reading fluency. The mean braille reading speed in congentially and early blind individuals is usually 80–120 words-per-minute [13,19–21] and some of them achieve reading speeds of 200 words-per-minute and more [13], a speed comparable with visual reading [35]. At the same time, the reading fluency of late-blind subjects is on average two to three times lower and some are unable to learn braille at all [4,13,20,22]. Some studies link this with lack of professional braille education specifically tailored to late blind, adult subjects [13] and a decrease in tactile acuity with age, which might make learning braille later in life increasingly difficult [4,7]. However, a large variance in braille reading fluency is also observed within congenitally/early blind and late blind populations [13,20,22]. This variance was shown to be related to inter-individual differences in perceptual and cognitive abilities, such as tactile acuity, phonological awareness and verbal short-term memory [33].

Given that several studies show the importance of tactile acuity for braille reading in blind subjects [6,7,33] and that sighted individuals have lower tactile acuity than the blind [3–7], we hypothesized that tactile acuity might be particularly important for sighted subjects to progress in braille reading. However, we found no correlation between the final braille reading speed and the tactile acuity on the reading finger. We also found no difference in final braille reading speed between subjects with the lowest tactile acuity and the rest of the group. It is possible that tactile acuity becomes critical at higher braille reading speeds. Alternatively, this relationship might have been obscured by other factors, for example differences in subjects’ motivation and persistence. Unfortunately, tactile acuity data was collected only at the end of the braille course, which makes impossible to test for a change in this measure, potentially induced by braille learning. Nevertheless, data collected at the end of the course shows that even subjects with low tactile acuity were able to read whole braille words. Thus, the spatial resolution of the sighted’s sensorimotor system is not a limiting factor for learning whole-word braille reading, at least at this learning stage.

### Learning braille in sighted adults and blind children: a comparison

After 9 months of training, our sighted subjects reached mean braille reading speed of 6 words-per-minute. This is arguably a very slow pace when compared to the final braille reading speed in the blind or standard visual reading speed in the sighted. However, the process of achieving high braille reading fluency in blind individuals is also very slow and arduous. Lorimer [27], in particular, reported that median braille reading speed for 8-year-old blind children is around 10 words-per-minute. Then, the average braille reading fluency improves very slowly, reaching a median of 40 words-per-minute at the age of 13. The annual increase in braille reading speed in blind children is thus 6 words-per-minute on average, and the pace of learning is relatively constant and linear. Emerson et al. [28] reported much higher reading fluency in blind children in the 1^st^ grade (34 words-per-minute). However, this high figure is perhaps an effect of a particularly early onset of braille education in this group–while reading rates of these blind children were collected for the first time in the 1^st^ grade, the authors reported that most subjects joined the study and started to learn braille in kindergarten or prekindergarten. Yet, the same authors also reported that the subsequent pace of improvements in braille reading speed in that group was on average 9 words-per-minute in each year. Both studies thus show that in blind children, the average pace of improvements in tactile reading fluency is equal to 6–9 words-per-minute in each year.

These results are comparable to data from our sighted subjects, who after 9 months of training reached braille reading speed of 6 words-per-minute. Although this comparison should be treated with caution, given differences in curriculum, workload, motivation and cognitive abilities among blind children and our subjects, it suggests that visual deprivation does not significantly increase the pace of achieving braille reading fluency. Thus, our results might suggest that experience-dependent mechanisms largely account for blind individuals’ proficiency in complex tactile skills, such as braille reading. We cannot exclude, however, the possibility that visual deprivation becomes critical at further stages of braille learning and higher braille reading speeds. One study, for example, shows that tactile processing is accelerated in congenitally blind braille readers relative to sighted subjects [2], which can be important at high braille reading speeds but not necessarily at the initial stage of learning. Given that the blind’s advantage was found both for the preferred reading finger and for the opposite index finger, the authors suggest that the origin of this perceptual enhancement might be deprivation-based rather than experience-dependent. They acknowledge, however, that generalization of perceptual tactile learning across hands and fingers [14,15,17,18] might be an alternative explanation for their result. Further studies are needed to resolve whether visual deprivation produces perceptual enhancements that cannot be achieved during prolonged and complex training.

## Conclusions

In summary, we show that most sighted adults can learn whole-word braille reading, given the right method and a considerable amount of motivation. Thus, the adult sensorimotor system can, to some level, adapt to very complex tactile tasks without visual deprivation. Given that pace of braille learning was similar among blind children and our sighted subjects, our results might suggest that experience-dependent mechanisms largely account for the blind’s proficiency in complex tactile tasks. This conclusion should be, however, treated with caution, as we cannot exclude the possibility that visual deprivation causes specific perceptual enhancements that become critical at high braille reading speeds.

## Supporting Information

S1 File Data from the study. This database includes demographic data, tactile braille word/letter scores for each subject and testing session, individual grating orientation thresholds and results of the test of visual reading in regular alphabet (number of words read in one minute and percent of correct answers in the comprehension test).
(XLSX)
